# Considering medical risk information and communicating values: A mixed-method study of women’s choice in prenatal testing

**DOI:** 10.1371/journal.pone.0173669

**Published:** 2017-03-29

**Authors:** An Chen, Henni Tenhunen, Paulus Torkki, Seppo Heinonen, Paul Lillrank, Vedran Stefanovic

**Affiliations:** 1 Aalto University, Institute of Healthcare Engineering, Management and Architecture (HEMA), Espoo, Finland; 2 Fetomaternal Medical Center, Department of Obstetrics and Gynecology, Helsinki University Hospital and University of Helsinki, Helsinki, Finland; Universita degli Studi di Roma Tor Vergata, ITALY

## Abstract

**Introduction:**

Nowadays, an important decision for pregnant women is whether to undergo prenatal testing for aneuploidies and which tests to uptake. We investigate the factors influencing women’s choices between non-invasive prenatal testing (NIPT) and invasive prenatal tests in pregnancies with elevated a priori risk of fetal aneuploidies.

**Methodology:**

This is a mixed-method study. We used medical data (1st Jan 2015-31st Dec 2015) about women participating in further testing at Fetomaternal Medical Center at Helsinki University Hospital and employed Chi-square tests and ANOVA to compare the groups of women choosing different methods. Multinomial logistic regressions revealed the significant clinical factors influencing women’s choice. We explored the underlying values, beliefs, attitudes and other psychosocial factors that affect women’s choice by interviewing women with the Theory of Planned Behavior framework. The semi-structured interview data were processed by thematic analysis.

**Results:**

Statistical data indicated that gestational age and counseling day were strong factors influencing women’s choice. Interview data revealed that women’s values and moral principles on pregnancy and childbirth chiefly determined the choices. Behavioral beliefs (*e*.*g*. *safety and accuracy*) and perceived choice control (*e*.*g*. *easiness*, *rapidness and convenience*) were also important and the major trade-offs happened between these constructs.

**Discussion:**

Values are the determinants of women’s choice. Service availability and convenience are strong factors. Medical risk status in this choice context is not highly influential. Choice aids can be developed by helping women to identify their leading values in prenatal testing and by providing lists of value-matching test options and attributes.

## Introduction

### Choices in prenatal testing

As the critical component of high-quality and evidence-based prenatal care, prenatal screening and testing helps pregnant women to establish a risk profile for their pregnancies and provides information for birth preparations and further decisions [1, 2]. Usually, prenatal screening and testing includes two stages. First, the risk of fetal aneuploidy is assessed with initial screening; then the screen-positive women are offered diagnostic testing to further evaluate or confirm aneuploidy [3, 4].

Screen-positive women have to decide whether to undergo further prenatal testing for fetal aneuploidies and which test to take. Advances in genomic technology increase women’s options in prenatal testing [5]. The commonly available tests include amniocentesis, chorionic villus sampling (CVS) and the non-invasive prenatal test (NIPT)[5]. Invasive procedures, amniocentesis and CVS, provide an accurate diagnosis but carry a miscarriage risk. In contrast, NIPT, which is based on next-generation sequencing of cell-free fetal DNA in maternal plasma to assess the most common fetal aneuploidies, only uses maternal blood samples [6]. The weakness of NIPT is its false positive rates, which, although very small, require confirmation of the result by invasive procedures [7].

As each test technique has its own strong and weak points, there is no universal consensus about which test to offer [8, 9]. In line with the aim of autonomous reproductive choice, leaving the choice to the women is the only solution [2, 9]. The widely accepted ideal is that prenatal test choices should be voluntary, autonomous and congruent with the parents’ values and preferences [10]. However, choice-making for further tests is not easy. Testing options force screen-positive women to make choices that are usually uncertain, difficult and even painful [1, 11]. Thus, it is highly important to offer appropriate choice aids that allow women to make truly autonomous choices in such context [12, 13]. So far there is still a lack of high-quality women-centered choice aids [10], the development of which requires exploring pregnant women’s reasoning and identifying the influential factors of women’s choice [10, 14].

### Recent studies on choice-making in prenatal testing

During the last decade, several studies have been performed on women’s choice in prenatal testing. Kenyon (2014) investigated clinical factors that potentially influence women’s uptake on NIPT as a second-tier test [15]. The study indicated that women who received abnormal screening results in the first trimester were more likely to choose NIPT than women receiving results in the second trimester, and that risk scores influenced the follow-up test choice. Manegold-Brauer et al. (2015) found that uptake of NIPT was influenced by economic factors and the reimbursement of costs by the insurance companies was a significant issue affecting women’s choice [16]. Lewis et al. (2014) explored the justifications for the hypothetical decisions on prenatal testing and they listed a number of women’s values, beliefs and attitudes associated with testing, for example, “not wanting to risk the safety of my baby”, “my wish to have as much information as possible about the baby” and “I felt I could cope raising a child with Down syndrome” [17]. In their latest study, Lewis et al. (2016) empirically assessed women’s experience of being offered NIPT and identified that reassurance was the main motivator for accepting NIPT [18]. Godino et al. (2013) systematically reviewed 11 studies from 2002 to 2012 regarding the factors influencing the uptake of invasive testing by women with advanced maternal ages [19]. They suspected that psychosocial factors, maternal age, ethnicity, involvement of the partner in the decision-making process, availability of prenatal tests and counseling services may influence women’s decisions.

Due to the large worldwide variety in testing and counseling protocols, generalization of the study results is not possible. First, there are different types of testing programs and reimbursement plans that create different choice situations. Kenyon (2014) studied NIPT as a second tier screening before diagnostic tests [15], while Chetty et al. (2013) studied it as an alternative to invasive tests [8]. Second, although NIPT has been used in the field of prenatal screening and testing for almost a decade, there is a limited number of studies to investigate women’s choices in actual clinical practice following the adoption of NIPT. Chetty et al. (2013) investigated women’s hypothetical attitudes on NIPT, but NIPT and invasive tests were not in equal standing [8]. Third, many studies focused on the acceptance or uptake of NIPT or invasive tests, but there is still insufficient experience on how women choose among the three test options: NIPT, CVS and amniocentesis after positive first or second trimester screening. Fourth, most studies employed either qualitative or quantitative methods, whereas multi-method studies are far and few.

In this study, we design a mixed-method study to better understand how women choose among prenatal tests—NIPT, CVS and amniocentesis—within the public maternal service system in Finland, where all the mentioned tests are offered free of charge to screen-positive women. Thus we can control for the monetary effects of the different tests on women’s choice-making. We aim to share our initial experience in this particular clinical setting and demonstrate the utilization of different testing methods.

## Methods

### Study context: Prenatal testing in the District of Helsinki and Uusimaa

This study was carried out at Fetomaternal Medical Center (FMC) which serves as a tertiary center for fetal medicine in the District of Helsinki and Uusimaa (HUS). HUS was the first Finnish public hospital to provide NIPT from the 1st of January, 2015, as an alternative to the invasive fetal diagnostics for women with the high-risk for fetal aneuploidy either *a priori* (maternal age > 40 years and first trimester combined screening unperformed for any reason and those with the common aneuploidy in the previous pregnancy), or abnormal result in the first trimester combined screening (risk for 21-trrisomy ≥1:250, fetal nuchal translucency 3–3.9 mm), or abnormal second trimester screening. In a few rare cases with the presence of two or more soft markers or fetal anomaly at the second trimester ultrasound for those who would not terminate the pregnancy under any circumstances, NIPT has been offered as an alternative to the invasive procedure. In such cases, the main goal was to exclude with high probability fatal 18-and 13-trisomies, in the presence of which the cesarean delivery would be performed only for maternal indications, if needed.

All women have been referred to an individual non-directive counseling provided by trained midwives at FMC informing women about chromosomal conditions, their individual risk scores, attributes of the three tests (reliability, miscarriage risk, feasibility regarding gestational age, test schedule, and waiting times for the results) and their comparisons and the service procedures (S1 Fig). Women were offered the choice between NIPT and invasive procedures. If the result of NIPT was abnormal, no further testing was offered except the standard second trimester genetic sonogram. In the cases of positive NIPT, post-testing counseling was offered regarding invasive diagnostic procedure and the further pregnancy management according to the results. Act on Termination of Pregnancy (TOP) in Finland allows TOP up to the gestational week 24. In case of abnormal NIPT, TOP may be performed exclusively after the confirmation of NIPT results by invasive procedure.

A crucial service availability issue is present in the study: the NIPT sample drawing service (blood test) is only offered on Mondays and Tuesdays at the HUS laboratory, while invasive tests and counseling service are available across all working days. Women who had the counseling on Wednesday/Thursday/Friday and chose NIPT had to come back for the blood test the following Monday/Tuesday. On average, turn-over time for NIPT was around two weeks while the results of qPCR for common trisomies were available within 3 days and the whole karyotype within 3 weeks on average. Blood samples for NIPT were sent to the USA for the analysis (The Harmony Prenatal Test by Ariosa Diagnostics).

### Study design

In this study, we focus on the group of women who were eligible for prenatal testing because of high risk detected by the first or the second trimester serum screening. This group accounted for the majority of women who were eligible for further testing. Other medical indications of high risk, including abnormal nuchal translucency, previous chromosomal problems and structural abnormality, have different risk indicators, with which the analysis will become complex. The counseling service for our study group of patients differs from those with slightly increased NT or those who opt for NIPT or invasive due to the a-priori risk (e.g. advanced maternal age, previous aneuploidy), so it is of great importance that all women in our cohort represent the same group. We conducted a mixed-method research involving quantitative data and qualitative data, which could lead to greater validity and rigor and provide a better understanding of the research problem than either research approach alone [20]. Many researchers have agreed that mixed methods approaches can be particularly useful in healthcare research that requires a broader range of perspectives to view the complexity in this field [21–23]. We followed the concurrent triangulation design [24], in which quantitative study and qualitative study were conducted at the same time period and the results were converged in the interpretation phase. This study was approved by HUS Ethical Committee (permission number: 220/13/03/03/2015).

### Quantitative study

We utilized FMC test choice database about women who participated in further testing between 1 January 2015 and 31 December 2015. FMC patient test record includes information about women’s choice on further tests, risk scores, maternal age, gestational age at counseling and the counseling date. The Chi-square test (or Fisher’s Exact Test, when appropriate) was used to compare differences in screening trimesters and counseling day (Monday and Tuesday = 1) among women who opted for NIPT, CVS and amniocentesis. One-way ANOVAs combined with post-hoc Scheffe tests [25] were used to detect the differences in maternal age and gestational age at the counseling among these women who chose different tests. Two multinomial logistic regressions, one with CVS group as the referent and the other with amniocentesis group as the referent, helped to identify the significant factors influencing women’s choices on the three tests, enabling the pair comparisons. Statistical analysis was performed using STATA 13. A p-value of <0.05 was used to establish statistical significance.

### Qualitative study

More detailed logic of women’s choice making was explored by interviewing those who participated in further testing because of the high risk revealed by the maternal serum screening. Midwives at the FMC participated in recruiting the interviewees after the pre-test counseling by presenting the “letter to patient”. All the informants voluntarily participating in the interviews have signed the written informed consent. We continued to conduct the interviews until we got the sample that could represent the whole population regarding the test method selections, NIPT and invasive tests. After 6 months of interviews, we deemed to have reached the data saturation point regarding a representative sample. Semi-structured interviews included open-ended questions about why and how the woman made choices in further testing. The concrete questions were developed from Theory of Planned Behavior (TPB) [26, 27], which helps to identify the determinants of women’s choice with three main constructs: behavioral beliefs, subjective norms and perceived behavioral control. Most of the interviews were conducted in Finnish and two in English. Interviews were tape recorded, transcribed, translated into English and imported into *Atlas*.*ti* qualitative data analysis software. Author AC and HT independently coded the data, identified the factors/items for each TPB construct (deductive analysis) and also tried to explore new constructs and corresponding factors/items (inductive analysis). Then the two authors discussed, reached an agreement and thematically integrated the data within the theoretical framework that was derived from TPB added with the unearthed constructs. We enhanced the validity of findings by organizing discussions in the research team, consulting the experts from delivery hospitals and maternal clinics, and getting feedbacks from relevant parties and professionals.

## Results

### Results from quantitative analysis

#### Group differences

During the study period (1st Jan 2015-31st Dec 2015), there were 254 women (62.5% of all participants) who participated in prenatal testing with abnormal serum screening result. NIPT was chosen by 186 women (73.2%), 39 (15.4%) chose CVS and 29 (11.4%) chose amniocentesis. Clinical characteristics of the study population with abnormal serum screening results and following the three further tests are provided in Table 1. The statistical test showed that on average, the NIPT group had a more advanced age than the two invasive test groups and the difference is significant (p = 0.015). The amniocentesis group had a significantly higher gestational age than the two groups (p<0.001). Women choosing NIPT were more likely to have counseling on Monday or Tuesday (p<0.001). Compared to amniocentesis choosers, CVS and NIPT choosers were more likely to have the first trimester screening (p<0.001, Fisher's exact). No women from the second trimester screening group chose CVS. Women choosing CVS had higher serum screening risk scores (p = 0.014).

**Table 1:** Clinical characteristics of women choosing NIPT, CVS and amniocentesis.

	Total population (n = 254)	NIPT (n = 186)	CVS (n = 39)	amniocentesis (n = 29)	*p* value (anova or chi square test)	*p* value (NIPT VS CVS)	*p* value (CVS VS amniocentesis)	*p* value (NIPT VS amniocentesis)
Maternal age, mean (SD)	35.7 (5.2)	36.2 (5.2)	35.2 (4.8)	33.3 (5.3)	0.015*	0.544	0.314	0.018*
Gestational age, mean (SD)	100.1 (12.9)	98.9 (11.5)	92.1 (4.3)	118.2 (12.5)	0.000**	0.002**	0.000**	0.000**
Counseling day								
Monday—Tuesday, n(%)	169 (66.5%)	139 (74.7%)	14(35.9%)	16 (55.2%)	0.000**	0.000*	0.135	0.029*
Wednesday—Friday, n(%)	84 (33.5%)	47 (25.3%)	24 (64.1%)	13 (44.8%)				
Trimester of screening								
the first trimester screening, n(%)	218 (85.8%)	169 (90.9%)	39 (100%)	10 (34.5%)	0.000**(0.000** Fisher’s exact)	0.050 (0.049* Fisher’s exact)	0.000**(0.000** Fisher’s exact)	0.000**(0.000** Fisher’s exact)
the second trimester screening, n(%)	36 (14.2%)	17 (9.1%)	0 (0.0%)	19 (65.5%)				
Serum screening risk score, mean (SD)	0.022 (0.041)	0.019 (0.035)	0.041 (0.068)	0.018 (0.021)	0.014**	0.137	0.054	0.863

*p<0.05;

**p<0.01

#### Influential factors

Multinomial logistic regressions reveal that gestational age and counseling day were the strong factors of women’s choice in prenatal testing, while other medical factors including the trimesters of screening and serum screening risk scores were not. Table 2 presents the RR ratios and confidence intervals (CI) of each factor. When compared to the women choosing NIPT, women with lower gestational age were more likely to choose CVS (p = 0.014) and women with higher gestational age were more likely to choose amniocentesis (p<0.001). Women coming to FMC for counseling service on Monday or Tuesday were more likely to choose NIPT over CVS (p<0.001) and amniocentesis (p = 0.028). S1 Table presents the result of statistical power analysis for the regression by Monte Carlo simulation [28]. We acknowledge the statistical power is not high for some variables (maternal age and serum screening risk score) with small effect sizes in our model. However, some of the power and effect sizes are at a good level (counseling day, screening trimester and gestational age). The dataset we used for this study has included all women participating in the further testing at FMC in 2015, i.e. the data of the whole population. During 2016, hospital made changes to the test service and test protocol offering invasive test and molecular karyotyping to women with fetal nuchal translucency ≥3.5mm and FTS risk ≥1:10. Therefore it is hard to combine the data of 2015 with that of following years.

**Table 2:** Factors influencing women’s choice in prenatal testing.

	Choosing NIPT vs Choosing CVS			Choosing Amniocentesis vs Choosing CVS			Choosing NIPT vs Choosing Amniocentesis		
Predictors	RRR	95(%) CI	p	RRR	95(%) CI	p	RRR	95(%) CI	p
Maternal age	1.06	0.99—1.14	0.125	0.97	0.86—1.10	0.628	1.09	0.99—1.20	0.089
Gestational age	1.09	1.02—1.17	0.014*	1.17	1.07—1.28	0.000**	0.93	0.88—0.99	0.013*
Counseling day									
—Monday or Tuesday = 1	4.47	2.04—9.85	0.000**	1.36	0.38—4.82	0.637	3.30	1.13—9.56	0.028*
—Wednesday, Thursday or Friday = 0	referent								
Screening trimester									
—The second trimester screening = 2	84183.89	0—(choosingCVS = 0)	0.984	230507.20	0 —(choosingCVS = 0)	0.983	0.37	0.07—1.92	0.235
—The first trimester screening = 1	referent								
Serum screening risk score	1.00	1.00—1.01	0.135	1.00	0.99—1.01	0.510	1.01	1.00—1.02	0.088

*p<0.05;

**p<0.01

### Results from interviews

We interviewed 26 women (S2 Table shows the participants’ information), of which 21 (80.8%) chose NIPT, 3 (14.3%) chose CVS and 2 (7.7%) chose amniocentesis. Table 3 displays the choice-influencing factors that were mentioned by the participants and organized within the framework of five choice-related constructs: values, behavioral beliefs, subjective norms and perceived choice control. It provides a picture of why women chose NIPT, CVS or amniocentesis and their concerns in the choice-making.

**Table 3:** Factors that affected women’s choice on further tests in terms of values, behavioral beliefs, subjective norms and perceived choice control.

Constructs	Chose NIPT (21)		Chose CVS (3)		Chose amniocentesis (2)		Trade-offs
	For NIPT	Against NIPT	For CVS	Against CVS	For amniocentesis	Against amniocentesis	
**Values**: moral principle or personal normative beliefs without the facts or counseling information	**Keep the baby unconditionally and safely obtain information for preparation (5)** *“I don't want to end the pregnancy for no matter what; I just wanted to know more about the baby's status and prepare the labor and further care*.*”* **Sequential choice, from mild to strong (5)** *“Probably there is nothing wrong*. *I don’t want to jeopardize the baby if there is nothing wrong*. *I want to take the stronger test later*.*”*	-	**Avoid having a sick baby (1)** *“We hope to have a healthy baby”*	-	**Uncertainty avoidance (1)** *“Uncertainty is the worst thing in life*.*”* **Avoid having a sick baby (1)** *“I do not want to have a sick baby*. *If I give a birth*, *definitively I want a healthy baby*.*”*	-	**Behavioral beliefs vs. perceived choice control (5)** *“For me the miscarriage is the bigger factor than the waiting time*. *Amniocentesis is faster but the miscarriage risk is the issue*.*”* **Behavioral beliefs vs behavioral beliefs (3)** *“We care more about the accuracy than the safety and the fear of the miscarriage risk*.*”* **Values vs behavioral beliefs (2)***“There is false negative risk*. *No further test if the result of NIPT is negative*. *But anyway*, *the end result for us is the same*. *We will keep the baby for no matter what*.*”* **Trade-offs between risks (1)** *“I think my risk score is low and the risk of miscarriage of invasive is about the same*. *I don't want to take the risk to lose the baby*. *Miscarriage from invasive tests is more likely than getting down baby*.*”*
**Behavioral beliefs**: advantages/disadvantages of and attitude toward different choice consequences	**Safety (20)** *“NIPT is not harmful to the baby*. *There is no miscarriage risk*.*”* **Sufficient accuracy (12)** *“NIPT is sufficiently accurate for my case*.*”*	**Accuracy (6)** *“NIPT is not as accurate and reliable as invasive tests*. *There is false negative risk*.*”*	**Accuracy (3)** *“CVS is reliable and provides certainty*.*”*	**Safety (3)** *“There is miscarriage risk in CVS*.*”*	**Accuracy (2)** *“I heard that amniocentesis is 100% sure*. *So I wanted to do that*.*”* **Ground for early abortion (1)** *“We cannot get abortion based on positive NIPT*. *I chose invasive test to be able to have the abortion as early as possible if the baby has problems*, *in order to avoid getting too attached to the baby*.*”*	**Safety (2)** *“There is an infection and miscarriage risk in amniocentesis*.*”*	
**Subjective norms**: social influence on choice	**Husband or partner (10)** *“My decision was supported by my husband*. *Our joint choice”* **Friend (1)** *“my friend recommended NIPT*.*”* **Medical staff (1)** *“I had a feeling that the midwife thought NIPT was better for my case*.*”*	**Husband or partner (1)** *“My husband preferred amniocentesis because the pregnancy has been already 16 weeks”* **Friend (1)** *“One of my friends might have had NIPT*. *But it does not influence my decision*, *because I was not sure whether it was the same situation*, *or she spoke about screening*, *not NIPT*.*”*	**Husband or partner (2)** *“My husband agreed with my choice*.*”*	-	**Extended family (1)** *“Amniocentesis is very common*. *One father's relative did amniocentesis before*.*”*	**Family (1)** *“My sister has different opinion but it didn't change my mind”*	
**Perceived choice control**: technical or practical issues related to the test process, and perceived ease or difficulty of going through the tests	**Easiness (11)** *“NIPT is just taking the blood sample*. *It is an easy procedure*.*”* **Comfort (7)** *“NIPT is not physically as uncomfortable as CVS*, *and I don't want the pain”* **Convenience (3)** *“I could take the test right away*. *After the counseling I can go straight to the blood test”* **Familiarity (2)** *“Taking the blood sample is familiar to me*.*”* **Controllability (1)** *“I feel NIPT process is more controllable than others”* **Medical fact (1)** *“NIPT is good for my case*, *because the down risk is small and other risk is very small*.*”*	**Rapidness (8)** *“I have to wait for two weeks to get the test result*. *It is slow*.*”* **Feasibility (1)** *“NIPT may fail due to the lack of fetal DNA in the maternal blood*.*”*	**Rapidness (3)** *“It is quick to get answer by CVS*.*”* **Convenience (1)** *“It was not convenient to come to the hospital again for the NIPT test on other day*. *CVS was offered on the same day of my counselling*.*”* **Feasibility (1)** *“CVS is feasible at this stage of my pregnancy*. *The timing is good*.*”*	**Comfort (2)** *“The feeling of needle going through the belly and placenta is not good”* **Easiness (1)** *“the procedure is not as easy as NIPT”*	**Rapidness (2)** *“After the test*, *the result comes in one week*, *faster than NIPT”*	**Controllability (1)** *“I was afraid of procedure*. *I would blame myself if I moved so as to cause problems to the test and the baby*.*”*	

#### Values

Half of our interviewees had clear life-guiding and option-unaffected values related to reproduction before participating in prenatal testing. Values include moral principles or personal normative beliefs without the facts or counseling information about the tests. The common values mentioned by our participants included “keep the baby unconditionally and safely obtain information for preparation”, “sequential choice, from mild to strong”, “uncertainty avoidance” and “avoid having a sick baby”. Five women who chose NIPT claimed that they would keep the baby in any condition and take the test to get more information about the baby’s status and prepare for the delivery and future care. Five women said they wanted to go through the testing sequentially starting from the less invasive option. These five women chose NIPT first and would go to do diagnostic testing depending on the NIPT result. Two women, one opting for CVS and the other choosing amniocentesis, suggested that they hoped to have a healthy baby and it would be hard for them to take care of a disabled child. One women chose amniocentesis and mentioned that she wanted everything to be controllable in her life, suggesting the desire to obtain certainty.

#### Behavioral beliefs

In the interviews, we asked women about their beliefs on the advantages/disadvantages of the tests they chose and explored their attitudes toward choice consequences. All interviewed women had their own understandings about the attributes (advantages or disadvantages) of their choice consequences based on the information given by counselors or from other sources. For NIPT choosers, safety (mentioned by 20 women), namely no miscarriage risk, was the first main factor for them to make the choice. Some NIPT choosers (12 women) claimed that the accuracy of NIPT was high or sufficient enough for them. Six NIPT choosers acknowledged or worried about NIPT being less accurate than other tests. For invasive tests choosers, accuracy (mentioned by 5 women) was the dominant advantage. One amniocentesis chooser emphasized the possibility of early TOP of the abnormal fetus, the other appreciated the relative safety of amniocentesis compared to CVS. Nevertheless, safety (mentioned by 5 women) was the main concern the invasive tests choosers had.

#### Subjective norms

The interviews revealed that the women’s choices in prenatal testing were quite individual. In such risk-involved situations, women rarely shared their experiences with other people except for the partners. They considered it as their own right or responsibility to make the decision. Husband or partner (mentioned by 13 women) was the main person involved in the women’s choice-making process. Twelve women said that they got support from their husbands for the decision or it was their joint decision. One woman said she insisted in her choice although her husband did not agree with her. Two women reported that they contacted with friends when making the choice. One woman followed her friend’s recommendation; one woman felt the friend’s choice was doubtful and she denied it affecting her choice. Two women informed thinking about their family/extended family when they made the decision. One woman said she followed what her relative did in the same situation, while another informant said she insisted in her choice despite her family not agreeing. Only one woman described having a feeling that the medical staff was inclined to recommend certain option and that her choice was influenced by that.

#### Perceived choice control

We used the perceived choice control construct to identify the perceived ease or difficulty of going through the tests, particularly the technical or practical factors considered by women with regard to the test process. The factors that facilitated the women to choose NIPT include easiness of the blood sample drawing procedure (mentioned by 11 women), less physical pain compared to the invasive tests (mentioned by 7 women), convenience of the testing after the pre-test counseling (mentioned by 3 women), familiarity of the blood sample drawing procedure (mentioned by 2 women), perceived controllable process (mentioned by 1 woman) and the low serum screening risk (mentioned by 1 woman). These women who chose NIPT were concerned about long waiting time for the test result (mentioned by 8 women) and the possible failure of the test (mentioned by 1 woman). Women chose invasive tests because of the rapidness to get the result (mentioned by 5 women), convenience of the testing after the pre-test counseling (mentioned by 1 woman), and the good timing or technical feasibility of the testing according to the gestational age (mentioned by 1 woman). The main concerns held by the invasive test chooser included the pain caused by the testing (mentioned by 2 women), the complexity of the procedure (mentioned by 1 woman) and the possible poor controllability in the process (mentioned by 1 woman).

#### Trade-offs

Since no test technique can have supreme values across all aspects, trade-offs commonly occurred as women made choice between tests, comparing the options in terms of test attributes and putting different weights/preferences on the conflicting attributes. Trade-offs were made between the attributes within the same construct or across different constructs. Some women clearly expressed the perceived conflicting attributes of the different tests and what attributes they cared more about. Two women said they insisted on their values and made the choice based on that even though the quality of other aspects might be lost. There was only one woman who mentioned about the risks and calculated and balanced different risks when making the choice.

In summary, from the interview data, we found that women’s values and moral principles on pregnancy and childbirth (*keeping the baby and safely obtaining information for preparation; sequential choice from the mild to the strong; uncertainty avoidance; avoid having a sick baby*) were the factors that predominantly determined their choices on further tests. Behavioral beliefs (*safety and accuracy*) and perceived choice control (*easiness*, *comfort*, *convenience*, *rapidness*, *feasibility*, *controllability and familiarity*) were mainly influencing their choices. Trade-offs between the factors were quite common. Most women made the decision by themselves, and choices were usually supported by the partners but not strongly influenced by other people.

## Discussion

The main observation of the study is the high uptake of the NIPT in our study group of women with high-risk for fetal aneuploidy (78.1%). Kenyon (2014) reported similar findings. He specifically compared the uptake of diagnostic testing before and after the introduction of NIPT and revealed the high acceptance of NIPT (70.9%) [15]. NIPT is still a relatively new technology in prenatal testing, but it is well received, as it is viewed as a safe step to further detect fetal aneuploidies.

Several studies have suggested that the relationship between maternal age and choice of further tests was significant, the older the women the more likely they were to choose NIPT [15, 29]. Therefore it is surprising that in the Finnish prenatal testing context, the regressions did not indicate that maternal age would be a significant factor for women’s choice among the three tests. We consider that the cut point between high and low maternal ages is not so clear for women, as more and more women have their first child after turning 35.

Kenyon (2014) suggested that the trimester of initial screening impacted women’s choice, speculating that selecting NIPT as a contingent screening test after the first trimester gives women plenty of time to follow-up with a diagnostic test, while selecting NIPT after the second trimester may give women less time to make decisions about the further step if that becomes necessary [15]. However, in our study trimester of initial screening is not a strong factor, because its effect on choice has been largely explained by gestational age at the counseling moment.

According to many studies medical indications and risk detected by the screening can guide women’s choice for further tests. Nicolaides et al. (2005) identified that the uptake rate of invasive testing significantly increased with higher estimated risk [30]. These results are not confirmed by our study, as traditional risk status was not a powerful factor influencing women’s choice. Most of the interviewed women did not talk about the medical risk or balance the risk scores in their assessment, which supports the results of the statistical analysis that the medical risk score is not a strong predictor of choice. Even with detailed counseling medical indications and risks are very demanding to comprehend for persons with no medical background. There are different scales in women’s minds for understanding the risks, thus women interpret the risk variedly and vaguely.

Our study indicates that when financial and reimbursement factors are removed and women are given equal counseling about the three prenatal tests, psychological and practical factors have stronger influencing power on women’s choice. First, choice is values-led. This construct that is not included in the theory of planned behavior but contributes to explain significant portions of variance in the choice, is values. Values refer to the overarching ethical, religious, political, or social principles that guide how an individual lives [31, 32], the feelings of personal responsibility regarding the performance of a given action [26], the situation-transcending fundamental judgment of what is most important to life [33] or the views about what is right and wrong [34]. In this study, the prominent values with regards to the choice for the further testing are reflected in several major themes: *keeping the baby and safely obtaining information for preparation; sequential choice from the mild to the strong; uncertainty avoidance; avoid having a sick baby*. Women choose NIPT if they insist in keeping the baby and safely obtaining information for preparation or taking sequential choice from the mild to the strong, which is in line with the findings of Gyselaers et al. (2015) that contingent NIPT screening is both clinically and economically beneficial [35]. Women choose invasive tests if they want to avoid uncertainty or having a sick baby. Women’s values, especially ethical beliefs, assumingly play a leading role in the decision [34]. Since no treatment is available for the defects detected by the test, women receiving a test offer are thought to be confronted with ethical questions about the values of a disabled life and the parental responsibilities for an affected fetus. It is a common observation that women directly associate testing decisions with potential decisions about TOP [12], which always arouses ethical judgment. We also notice in our study that when women’s values are clear, the choice between tests can be determined straightly, easily and with less choice burden.

Second, choice is preference-sensitive. Key attributes of the test consequences—safety, accuracy and ground for abortion strongly influence women’s choice. Situation-specific preferences on the key attributes of the tests are congruent with the personally insistent situation-transcending values (e.g. women holding the moral principle of keeping the baby prefer the safer test). To make the choice, women combined their values with their beliefs about the options [36]. Once the values are clear, women would prefer the tests with the consequences mostly meeting with the values.

Third, choice is practice-considered. When making choices, women use self-reflection, image the possible procedures and mentally go through testing process in order to perceive the easiness or difficulty of it. Anticipated service experience in the procedure: easiness, rapidness, physical comfort, controllability and familiarity influence the choice. Technical feasibility/test timing, service availability and convenience are highly important factors influencing women’s choice, which is revealed by both quantitative and qualitative study. When women chose the tests, they considered the test timing and chose the test suitable to their gestational age at the pre-test counseling moment. Women strongly prefer the test that could be performed at the same day as the counseling service. This is supported by the study of Silcock et al (2014) in which women expressed a strong preference for testing on the same day as the pre-test counseling [6]. We observed that women would rather have a more risky test on the same day than return for a safer test on a separate day. This suggests that women wish to minimize the waiting times, as they tend to feel highly anxious about the results and medical risk information is not as straightforward to understand as the service practicalities. The anxiety of waiting for the test and result can be larger than the fear of miscarriage and complications related to the invasive tests.

Fourth, choice is made by trade-offs. Presentation of the conflicting attributes of tests leads to ambivalence and forces women to think about and make trade-offs. Women decide about prenatal testing by waveringly putting different weights on test attributes and balance the conflicting ones by gradually clarifying what is more important or what is unreplaceable. Once the reproduction-related values are certain, women have less hesitations and stress in making the choice.

For the clinical practice, to develop prenatal testing service in Finland and elsewhere, our results are useful in organizing tests and designing counseling, choice aids and other communications with patients. Our study’s first suggestion is that test counselors should effectively explore women’s supreme values that could determine the choice rather than simply consider their medical status and preferences for particular outcomes and practice [12]. Caregivers should provide the opportunity for the women to express, discuss and clarify their values, helping women to assess the meaning of testing within their life principles and then reduce decisional conflicts in choice-making [37]. Second, women-friendly choice environment should be built, which allows women to compare the information and make trade-offs between conflicting attributes without much cognitive and emotional burden. Appropriate methods should be employed to present medical information in a more concrete, visualized, comparable and understandable way. Third, if the objective is to help women to focus more on the medical risk, to make test attribute-based choices and not be highly influenced by the convenience of a test, test providers should strive for removing the restrictions related to service availability and convenience, e.g. provide tests equally in the same days.

**Fig 1:**
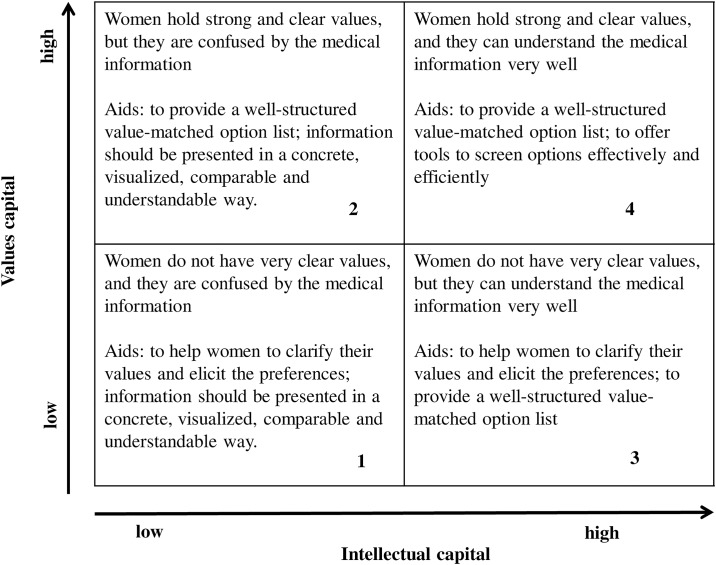
Women segmentation in prenatal testing based on values capital and intellectual capital.

Variations in the degree of consumer activeness and choice capabilities across patient subgroups have been investigated in healthcare [38–40]. Users of health services do not have an equal capital or capability to make informed and rational choices on their own. One type of aid cannot suit all patients. In order to provide patient-centered choice aids, service providers (e.g. choice counselors) should consider the varying capabilities, segment the patients and employ proper strategies or methods to assist patients’ choice exercises [41]. For aiding women’s choice-making in prenatal testing, women at pre-test counseling can be segmented into four types based on the two dimensions: *intellectual capital*, referring to the knowledge in medicine-related fields, the ability to understand and process information and the articulation in expressing preferences [41], and *values capital*, referring to the clearness and rootedness of values that women hold for life. Fig 1 presents the four segments and the specific choice aids for each group.

## Conclusion

Our study comprehensively explored the potential factors that may influence women’s choice in prenatal testing, especially in situations involving an elevated risk. We discovered that values are the determinants of women’s choice. Both quantitative and qualitative study indicated that technical feasibility and service convenience were strongly affecting women’s choice on test methods. Women considered their gestational ages, chose the tests based on the test performance time and preferred to have test on the same day as pre-test counseling. Our findings are directly useful in designing choice aids and counseling structures that alleviate the burdensome elements of prenatal testing decisions.

A lot more can be done to better understand women’s choice and reap the benefits of having different prenatal testing methods to choose from. This study can be extended by including women with other medical indications, e.g. abnormal nuchal translucency. A longer-term analysis including women with more varied medical indications would provide a broader view on women’s choice in a clinical setting. A wider database is also needed to improve the regression model. We have to acknowledge that the women who accepted to participate in our interview have positive attitude towards research and therefore they most likely have higher education background. This is definitely a limitation of the qualitative study with regard to generalization of the results. We aim to improve the study by conducting survey that is distributed widely among women with different educational levels and this way we try to solve the problem of selection bias present in this study. Surveys can help to further explore and evaluate psychosocial factors and the weights women put on different factors.

## Supporting information

S1 Fig Service flowchart of prenatal testing at FMC 1.1.2015–31.12.2015. (TIF)

S1 Table The result of statistical power analysis for the main regression by Monte Carlo simulation. (DOCX)

S2 Table Summary of interview sampling. (DOCX)
