# Supplementary figures and images for: Considering medical risk information and communicating values: A mixed-method study of women’s choice in prenatal testing
Source: PLoS One. 2017 Mar 29;12(3):e0173669. doi: 10.1371/journal.pone.0173669 (PMC5371284; doi:10.1371/journal.pone.0173669)

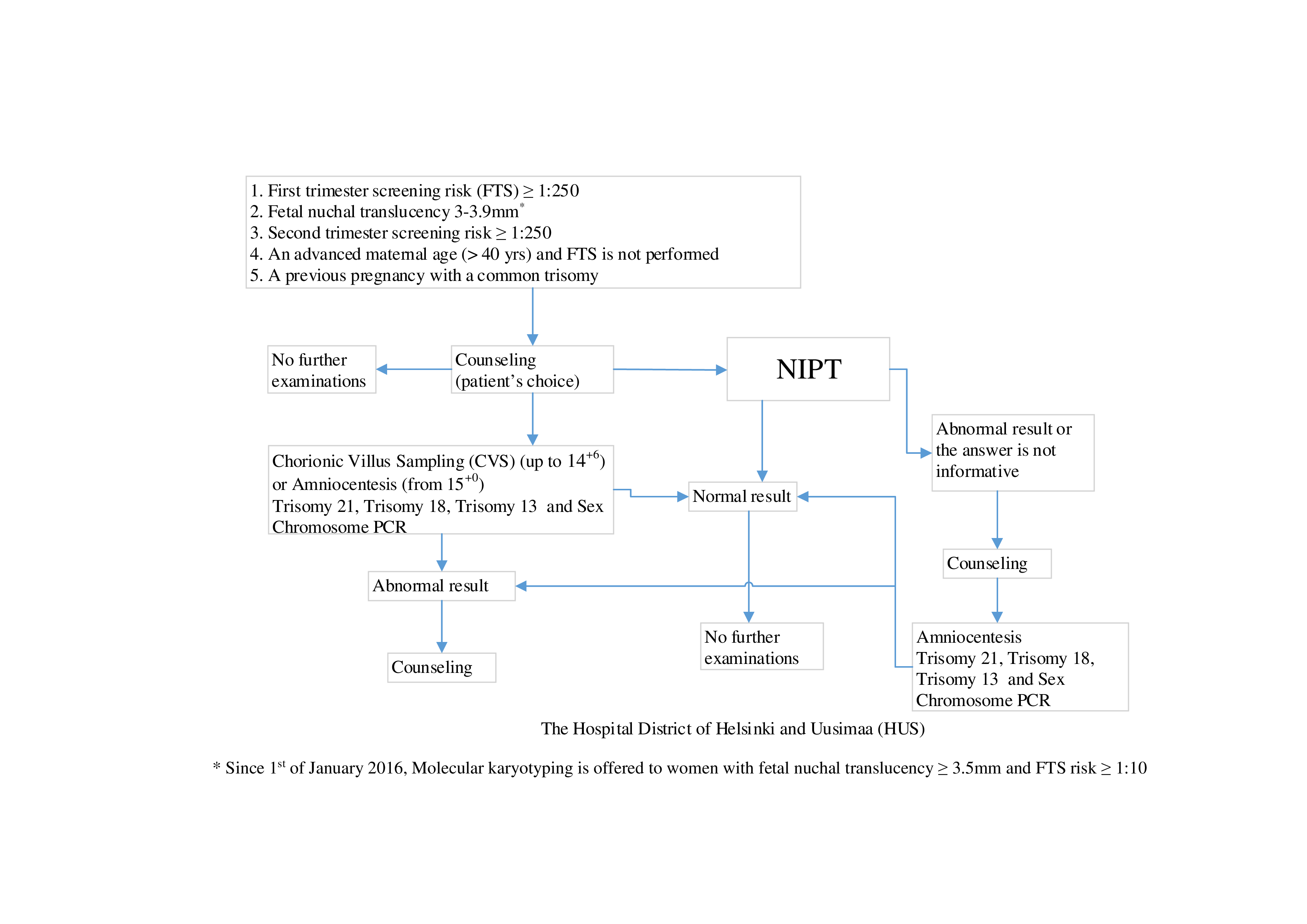

Supplement: S1 Fig — (TIF) [file pone.0173669.s001.tif]
