# Supplementary material for: Considering medical risk information and communicating values: A mixed-method study of women’s choice in prenatal testing
Source: PLoS One. 2017 Mar 29;12(3):e0173669. doi: 10.1371/journal.pone.0173669 (PMC5371284; doi:10.1371/journal.pone.0173669)
Supplement: S1 Table — (DOCX) [file pone.0173669.s002.docx]

**S1 Table. The result of statistical power analysis for the main regression by Monte Carlo simulation**

Observation 254, simulation 1000

|  | Maternal age | Gestational age | Counseling day | Screening trimester | Serum screening risk score |
| --- | --- | --- | --- | --- | --- |
| Choosing NIPT vs Choosing CVS | 354 (effect size 0.059) | 342 (effect size: 0.090) | 1000 (effect size: 1.499) | 1000 (effect size: 11.341) | 198 (effect size 0.006) |
| Choosing Amniocentesis vs Choosing CVS | 237 (effect size: - 0.029) | 575 (effect size: 0.158) | 999 (effect size: 0.305) | 1000 (effect size: 12.348) | 166 (effect size: 0.003) |
