# Supplementary material for: Considering medical risk information and communicating values: A mixed-method study of women’s choice in prenatal testing
Source: PLoS One. 2017 Mar 29;12(3):e0173669. doi: 10.1371/journal.pone.0173669 (PMC5371284; doi:10.1371/journal.pone.0173669)
Supplement: S2 Table — (DOCX) [file pone.0173669.s003.docx]

**S2 Table. Summary of interview sampling**

| **Participant No.** | **pre-test counselling choice: NIPT, CVS or amniocentesis** | **pregnancy weeks at counselling** | **trimester of screening** | **risk score** | **counselling day** | **Age** | **Education (highest diploma)** | **times of pregnancy** |
| --- | --- | --- | --- | --- | --- | --- | --- | --- |
| 1 | NIPT | 17^+1^ | 2 | 1:250 | Tuesday | 35 | doctor | 2 |
| 2 | NIPT | 14^+1^ | 1 | 1:250 | Tuesday | 27 | polytechnic bachelor | 1 |
| 3 | NIPT | 13^+4^ | 1 | 1:221 | Monday | 45 | master | 5 |
| 4 | NIPT | 14^+0^ | 1 | 1:139 | Tuesday | 39 | polytechnic bachelor | 4 |
| 5 | NIPT | 13^+1^ | 1 | 1:100 | Tuesday | 40 | master | 1 |
| 6 | CVS | 12^+4^ | 1 | 1:130 | Tuesday | 38 | polytechnic bachelor | 4 |
| 7 | NIPT | 13^+6^ | 1 | 1:142 | Monday | 37 | university bachelor | 2 |
| 8 | NIPT | 13^+0^ | 1 | 1:203 | Tuesday | 37 | polytechnic bachelor | 5 |
| 9 | NIPT | 13^+5^ | 1 | 1:40 | Monday | 41 | doctor | 3 |
| 10 | NIPT | 12^+4^ | 1 | 1:188 | Tuesday | 43 | polytechnic bachelor | 3 |
| 11 | NIPT | 14^+0^ | 1 | 1:199 | Friday | 40 | master | 1 |
| 12 | NIPT | 13^+5^ | 1 | 1:162 | Friday | 35 | polytechnic bachelor | 2 |
| 13 | NIPT | 15^+2^ | 1 | 1:115 | Monday | 37 | polytechnic bachelor | 2 |
| 14 | NIPT | 13^+6^ | 1 | 1:223 | Tuesday | 33 | master | 1 |
| 15 | NIPT | 12^+6^ | 1 | 1:125 | Tuesday | 26 | secondary education | 3 |
| 16 | Amniocentesis | 18^+2^ | 1 | 1:48 | Wednesday | 35 | polytechnic bachelor | 1 |
| 17 | CVS | 14^+0^ | 1 | 1:30 | Thursday | 39 | master | 3 |
| 18 | NIPT | 13^+4^ | 1 | 1:228 | Tuesday | 37 | polytechnic bachelor | 3 |
| 19 | NIPT | 12^+4^ | 1 | 1:91 | Tuesday | 27 | master | 1 |
| 20 | NIPT | 13^+3^ | 1 | 1:172 | Monday | 25 | university bachelor | 2 |
| 21 | NIPT | 18^+5^ | 2 | 1:240 | Monday | 39 | bachelor | 3 |
| 22 | Amniocentesis | 14^+1^ | 1 | 1:42 | Tuesday | 35 | doctor | 1 |
| 23 | NIPT | 17^+0^ | 2 | 1:210 | Tuesday | 34 | polytechnic bachelor | 6 |
| 24 | NIPT | 14^+5^ | 1 | 1:244 | Friday | 36 | master | 1 |
| 25 | CVS | 12^+5^ | 1 | DS 1:30; Edwards 1:52 | Monday | 35 | master | 2 |
| 26 | NIPT | 13^+5^ | 1 | 1, 1:140 | Tuesday | 30 | master | 2 |
